# Racial and Ethnic Representation in Preventive Intervention Research: a Methodological Study

**DOI:** 10.1007/s11121-023-01564-8

**Published:** 2023-06-29

**Authors:** Pamela R. Buckley, Velma McBride Murry, Charleen J. Gust, Amanda Ladika, Fred C. Pampel

**Affiliations:** 1 Institute of Behavioral Science, University of Colorado Boulder, Boulder, USA; 2 Departments of Health Policy & Human and Organizational Development, Vanderbilt University Medical Center and Vanderbilt University, Nashville, USA

**Keywords:** Clearinghouse, Registry, Racial equity, Evidence-based intervention, External validity, Diversity, Generalizability

## Abstract

Individuals who are Asian or Asian American, Black or African American, Native American or American Indian or Alaska Native, Native Hawaiian or Pacific Islander, and Hispanic or Latino (i.e., presently considered racial ethnic minoritized groups in the USA) lacked equal access to resources for mitigating risk during COVID-19, which highlighted public health disparities and exacerbated inequities rooted in structural racism that have contributed to many injustices, such as failing public school systems and unsafe neighborhoods. Minoritized groups are also vulnerable to climate change wherein the most severe harms disproportionately fall upon underserved communities. While systemic changes are needed to address these pervasive syndemic conditions, immediate efforts involve examining strategies to promote equitable health and well-being-which served as the impetus for this study. We conducted a descriptive analysis on the prevalence of culturally tailored interventions and reporting of sample characteristics among 885 programs with evaluations published from 2010 to 2021 and recorded in the *Blueprints for Healthy Youth Development* registry. Inferential analyses also examined (1) reporting time trends and (2) the relationship between study quality (i.e., strong methods, beneficial effects) and culturally tailored programs and racial ethnic enrollment. Two percent of programs were developed for Black or African American youth, and 4% targeted Hispanic or Latino populations. For the 77% of studies that reported race, most enrollees were White (35%) followed by Black or African American (28%), and 31% collapsed across race or categorized race with ethnicity. In the 64% of studies that reported ethnicity, 32% of enrollees were Hispanic or Latino. Reporting has not improved, and there was no relationship between high-quality studies and programs developed for racial ethnic youth, or samples with high proportions of racial ethnic enrollees. Research gaps on racial ethnic groups call for clear reporting and better representation to reduce disparities and improve the utility of interventions.

**Supplementary Information:**

The online version contains supplementary material available at 10.1007/s11121-023-01564-8.

Populations presently identified as racial ethnic minoritized groups in the United States (i.e., individuals who are Asian or Asian American, Black or African American, Native American or American Indian or Alaska Native, Native Hawaiian or Pacific Islander, and Hispanic or Latino, herein referred to as “racial ethnic” groups) are struggling through a syndemic, or multiple pandemics simultaneously (Lee et al., 2022). First, because racial ethnic groups disproportionately represented essential workers and lacked equitable access to resources that could mitigate exposure risk, COVID-19 brought racial and ethnic inequities to the forefront of public health, exacerbating discrepancies that already existed (Murry et al., 2022). Second, these discrepancies are rooted in manifestations of systemic, structural, and institutional racism that have resulted in a wide range of injustices, such as failing public school systems, unsafe neighborhoods, and unequal access to employment opportunities. In addition, residential areas where predominantly racial ethnic populations live have a disproportionate number of liquor stores (Lee et al., 2020), and residents are more likely to live in communities characterized as food and pharmacy deserts (Guadamuz et al., 2021). These systemic and structural discrepancies create environmental injustices associated with increased mental and behavioral problems and escalated risk for early onset of numerous chronic diseases (Lee et al., 2022). And third, racial ethnic residents are more likely to live near toxic waste facilities and more likely to die from exposure to pollutants (Mascarenhas et al., 2021; Woo et al., 2019). They are also particularly vulnerable to the greatest impacts of climate change wherein the most severe harms fall disproportionately upon underserved communities, whose members are least able to prepare for, and recover from, heat waves, poor air quality, flooding, and other extreme environmental impacts (EPA, 2021).

While there is an urgent need for system and structural level changes to address these pervasive syndemic conditions, more immediate efforts may be undertaken through preventive intervention research that demonstrate effective strategies for improving health and well-being. Increased visibility of racial and ethnic health disparities during COVID-19 presented a pressing need for the prevention science field to question the extent to which preventive interventions are designed to address issues of inequities adequately and sufficiently. Further, Murry and colleagues (2022) challenged the field of prevention science to deliberately design, develop, and implement preventive intervention programs to promote equitable health outcomes for racial and ethnic youth, families, and communities. Doing so, however, requires identifying preventive interventions that harness the strengths and cultural assets of racial and ethnic families, youth, and communities (Murry et al., 2018). Such approaches should be: (1) evidence based (i.e., show impact from evaluations with high internal validity; Steeger et al., 2021); (2) scalable (Buckley et al., 2020); and (3) culturally appropriate or contextualized through the perspectives of the beneficiaries and considering a social, cultural, and economic framework (Jackson, 2009; Murry et al., 2022). This challenge served as the impetus for the current study, as we sought to systematically review the inclusion of racial and ethnic groups in preventive intervention research, in which we conjectured there would be a lack of representation - as is well documented in medical research (Fisher & Kalbaugh, 2011; Turner et al., 2022). A critical evaluation of this omission in the prevention science field, however, has not been undertaken - though some research examining psychosocial interventions (broadly defined as non-pharmacological interventions focused on psychological or social factors) with documented effectiveness among racial ethnic youth has been conducted (see Pina et al., 2019). We used data from *Blueprints for Healthy Youth Development* (herein referred to as Blueprints; https://www.blueprintsprograms.org/), which provides an online registry of evidence-based preventive interventions that prevent or reduce the likelihood of antisocial and violent behavior and promote a healthy course of youth development and adult maturity (Mihalic & Elliott, 2015). Examining studies recorded in the Blueprints database, we reviewed the size, nature, and scope of extant research involving representation of racial and ethnic groups in preventive intervention research. In addition, following guidance from evidence standards for high-quality research establishing an intervention as efficacious or effective and ready for scale-up endorsed by the prevention science field (Gottfredson et al., 2015), we also examined setting (i.e., locale where the study was conducted) and additional sociodemographic factors. Our objectives were to (1) inform decision-making regarding the generalizability of evidence-based youth preventive interventions and (2) identify additional considerations of importance in guiding preventive intervention research, such as cultural adaptation and responsiveness (Thier et al., 2020). We consider implications of study findings for research, policy, and practice with a specific focus on potential inferences for future metrics to guide the prevention science field.

## Race and Ethnicity Reporting in Research

The inclusion of racial and ethnic participants in clinical trials is an important topic in public health discussions because doing so addresses critical issues for equity and equality that will in turn eliminate health disparities (Fisher & Kalbaugh, 2011). Turner et al. (2022) analyzed the reporting of race and ethnicity enrollment in trials of medical treatments, including drug efficacy studies, using all U.S. trials in the ClinicalTrials.gov online registry from 2000 to 2020 and found that, historically, U.S. trials under-enroll racial and ethnic groups and frequently do not report the race and ethnicity of enrolled participants at all. Meanwhile, Polo et al. (2019) examined trends over a 36-year period (from 1981 to 2016) in the reporting and representation of various sample demographic characteristics of trials evaluating psychotherapy and psychosocial treatments for clinical depression and found that the underrepresentation of racial and ethnic youth in these types of trials remains problematic. Though reporting improved over time, both studies concluded that the paucity of diversity has generated a data gap that skews evidence towards treatments with understudied efficacy and safety for racial ethnic populations.

The important sensitivities and controversies related to reporting of race and ethnicity noted in health research (see Flanagin et al., 2021) also apply to the prevention science field. Similar to medical trials, there are major gaps in research on racial and ethnic groups that impede effectiveness of preventive interventions, including insufficient attention to protective processes that prevent and avert risk (Murry et al., 2018), discounting input from beneficiaries representing diverse communities (Supplee & Meyer, 2015), and overlooking crucial information about how to effectively translate or adapt interventions that primarily target White populations for implementation among racial and ethnic populations (Rousseau & Gunia, 2016). Despite inclusion of racial ethnic populations, programs validated with largely White samples are often proposed to be recommended for all (Pina et al., 2019), which heightens external validity concerns about widely disseminated treatments tested for one group but exported, perhaps uncritically, to others (Thier et al., 2020).

## Race and Culture

Scientists have come to the consensus that race is not biological; it is socially constructed (Ioannidis et al., 2021). In general, ethnicity has historically referred to a person’s cultural identity (e.g., language, customs, and religion) and, while all groups have ethnicities (e.g., Caribbean, creole-speaking Blacks), race is characterized as broad categories of people that are divided arbitrarily but based on ancestral origin and physical characteristics. Though race and ethnicity have no biological meaning, the terms have important - albeit contested - social meanings (Flanagin et al., 2021). Culture can be viewed as the totality of a group’s knowledge, often transmitted from elders to children. “Having a culture” means that members share a collective system of values, beliefs, expectations, and norms, including traditions and customs, as well as established social networks and standards of conduct that define them as a cultural group (Barrera et al., 2013, 2017).

Adaptation occurs as preventive interventions move from efficacy trials, in which program developers typically supervise implementation, to effectiveness trials where developers are less involved (Gottfredson et al., 2015), and into the dissemination phases of the evidence continuum, characterized as exploration, preparation, implementation, and sustainment (Brown et al., 2017). Research on cultural adaptation, however, often equates “culture” with “racial and ethnic group status.” Such a focus may be derived from assumptions of homogeneity, without regard for heterogeneity of subgroups within a larger racial ethnic group based on nationality, socio-economic status, religious background, geographic residence, immigration status, and other issues that complicate “cultural” adaptation (Barrera et al., 2013).

## Standards of Evidence in Prevention Science

Preventive interventions serve diverse populations in terms of race, ethnicity, culture, and other sociodemographic factors. In disseminating empirical evidence to policy and practice, questions are continually raised about the extent to which the results of the available impact trials can be generalized to new populations, settings, and points in time. As decision-makers choose among a plethora of preventive interventions, a key element of their decision is whether or not a given program will work in their community, which might be different from the population included in the impact trial (Supplee & Meyer, 2015).

Guidelines (Gottfredson et al., 2015; Steeger et al., 2021) and checklists (Grant et al., 2018; Higgins et al., 2011; Schulz et al., 2010) exist for assessing threats to the internal validity of trials (i.e., the ability to make causal inferences), but these tools do not provide information on the context and potential applicability of research to local settings. Equally important is evaluating external validity, since the concerns of practitioners and policymakers relate to applicability of findings from preventive intervention trials (Bryan et al., 2021; Supplee & Meyer, 2015). External validity is also increasingly salient as developers of preventive interventions wrestle with implementation barriers (Walker et al., 2017). To minimize the risk of over- or under-ascribing an intervention’s utility to be scaled up, standards for the prevention science field require specifying target populations and settings and identifying for whom interventions work and under what conditions (Gottfredson et al., 2015). Even so, the prevention science field has not endorsed guidelines for assessing external validity, making it difficult to support decision-makers in this respect (Supplee & Meyer, 2015).

## Current Study

This paper used the Blueprints database to conduct a methodological study (i.e., a study that evaluates the design, conduct, analysis and/or reporting of other studies; Lawson et al., 2020) of race and ethnicity reporting and representation in preventive intervention research. We tracked reporting of gender, economic disadvantage, and geographic location, but focused particularly on the race and ethnicity of participants. The decision to use Blueprints was motivated by its range of youth behavioral outcomes representing multiple disciplines (e.g., criminal justice, child welfare, public health, mental health, education, and labor/employment) and our unrestricted access to the database. To our knowledge, the only prior research resembling our study synthesized biomedical/health (Turner et al., 2022) or clinical-level depression (Polo et al., 2019) outcomes of trials predominantly focused on adults and solely conducted in the United States. The present study examined reports and refereed journal articles of evaluations completed both within and outside the U.S. We included prevention interventions primarily focused on youth and evaluated using randomized control trials (RCTs) or quasi-experimental design studies (QEDs) encompassing a range of outcomes relevant to the prevention science field. In a review of evaluations entered into the Blueprints database over the past decade (2010–2021), our primary research questions included: (1) How prevalent were culturally tailored preventive interventions (i.e., ones developed for specific populations)? (2) What percent of evaluation studies reported sample characteristics? and (3) How well represented were racial and ethnic groups in samples of evaluation studies? We posed three secondary questions: (1) Has reporting of race, ethnicity, and sociodemographic characteristics improved over time? (2) Was reporting related to study quality (i.e., methodological soundness and beneficial effects)? and (3) Was study quality related to culturally tailored programs?

## Methods

### Eligibility Criteria

We restricted our sample to interventions with a study published between 2010 and 2021 in the Blueprints database (see Supplemental Panel S1 for a description of inclusion criteria). The 11-year time span allowed for inclusion of a large sample of preventive interventions while also focusing on relatively recent research that incorporated up-to-date initiatives of the registry. Before 2010, the more narrowly defined focus of Blueprints on delinquency and drug use limited the coverage of programs and prevented us from extending the study period any farther back.

### Search Strategy

We relied on Blueprints’ systematic search process to select studies, an approach that reduces bias in systematic reviews because it entails gathering and reviewing all relevant research (Wilson, 2009). See Supplemental Panel S2 for a description of the search strategy and Online Resource Table 1 for a listing of the search terms clauses used to locate studies.

**Table 1:** Descriptive results (sample of studies conducted in the United States)

	***N* (proportion)**
Interventions developed for a specific population (total *n* = 583):*	
Race	
Asian or Asian American	2 (.00)
Black or African American	12 (.02)
Native American or American Indian or Alaska Native	5 (.01)
Native Hawaiian or Pacific Islander	1 (.00)
White	0 (.00)
Ethnicity	
Hispanic or Latino	24 (.04)
Gender	44 (.08)
Economic disadvantage	32 (.06)
Location	
Rural	3 (.01)
Urban	10 (.02)
No specific group	455 (.78)
Studies that reported sample distribution of (total *n* = 583):	
Race	450 (.77)
Ethnicity-Hispanic or Latino	375 (.64)
Gender	509 (.87)
Economic disadvantage	168 (.29)
Location (rural, urban)	426 (.73)
Sample statistics for studies that report characteristic:	
Race (*n* = 450)	**Mean (SD)**
Asian or Asian American	.03 (.09)
Black or African American	.28 (.29)
Native American or American Indian or Alaska Native	.02 (.09)
Native Hawaiian or Pacific Islander	.00 (.00)
White	.35 (.29)
Multi-racial/biracial (must be specified this way)	.01 (.05)
Not specified	.31 (.26)
Ethnicity-Hispanic or Latino (*n* = 375)	.32 (.27)
Gender-female (*n* = 509)^a^	.50 (.22)
Economic disadvantage (*n* = 168)	.66 (.25)
Location (*n* = 426)	
Rural	.31 (.46)
Urban	.90 (.30)

^*^Percentages add to more than 100, as interventions may target multiple groups. ^a^Four studies reported another category for persons of nonbinary gender, which averaged .03% of sample participants

### Sample

**Fig. 1:**
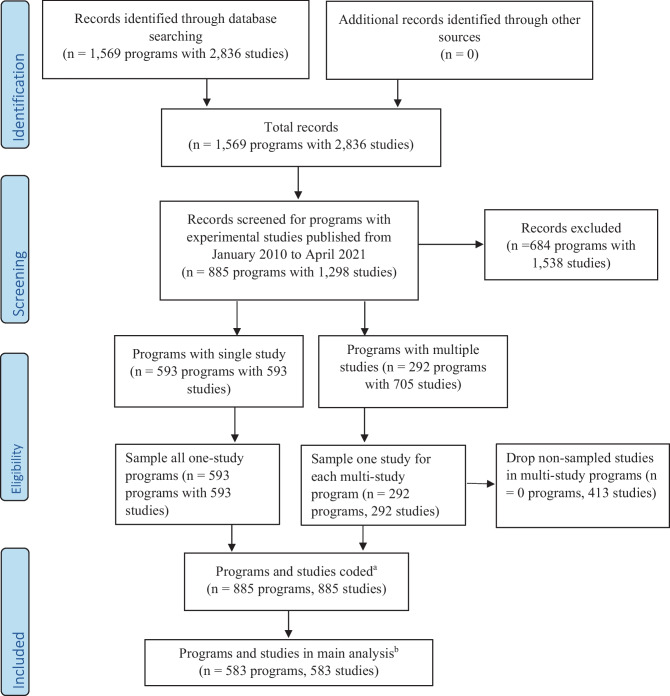
Flow diagram of systematic review based on PRISMA 2009 (Mohr, 2009). Notes: Blueprints-Blueprints for Healthy Youth Development, an online registry of preventive interventions (https://www.blueprintsprograms.org/). ^a^Studies conducted in the United States and outside the United States (mostly Europe, Australia, or New Zealand). ^b^Studies conducted only in the United States

Impact studies (i.e., research determining the efficacy or effectiveness of a preventive intervention or strategy; Gottfredson et al., 2015) comprised our sample, which came from preventive behavioral programs and associated evaluations of the programs contained in the Blueprints database (Fig. 1). As of April 2021, when a list of existing studies was created for coding and analysis, the Blueprints database had 1,569 programs that were added since 1996 (the registry’s inception). Nested within the 1,569 programs were 2,836 program evaluation studies. Selecting programs with studies having a report published between 2010 and 2021 reduced the sample to 885 programs with 1,298 studies. Most programs (*n* = 593, 67%) had only one study, but for the other 33% (*n* = 292) with multiple studies, we randomly selected one study to represent the program, thus creating a sample of 885 programs with one study each. We then coded each program and the representative evaluation of that program. Of these, 583 (66%) were conducted in the United States and comprised the main analysis sample, since U.S. Census codes (described below) were used to examine our research questions.

### Development of Coding Instrument

We drew from procedures adopted by other registries to construct and refine our codes (Lindsay et al., 2019; see Online Resource Table 2). First, we coded for whether the program was specifically developed for certain populations (i.e., culturally tailored), meaning services were developed for and provided in a manner that was responsive to race, ethnicity, gender, economic disadvantage, and/or geographic location. Second, we coded sample-level characteristics. We detail our extraction approach in Supplemental Panel S3. Briefly, we selected enrollment data for five racial categories to align with US Census coding operations (Asian; Black; Native American; Native Hawaiian or Pacific Islander and White) and treated ethnicity (coded as Hispanic or Latino) as separate from race (Humes et al., 2011). The codes for gender were male, female, and persons of nonbinary gender. For economic disadvantage, we considered the proportion of the sample that fell within the federal government’s poverty level as measured by different metrics, such as the percentage of students that qualified for free or reduced-price lunch (a proxy in education research for families that are low-income).

**Table 2:** Results of time-trend analysis (studies conducted in the United States)

**Outcomes**	**Coefficient for year of publication**	***p***
Interventions developed for a specific population (*n* = 583):*		
Race		
Asian or Asian American	.22	.438
Black or African American	− .25	.048
Native American or American Indian or Alaska Native	.08	.628
Native Hawaiian or Pacific Islander	.31	.471
White	–-	–-
Ethnicity		
Hispanic or Latino	− .01	.887
Gender	− .02	.790
Economic disadvantage	.04	.581
Location		
Rural	− .16	.500
Urban	− .13	315
No specific group	.01	.784
Studies that reported sample distribution of (*n* = 583):*		
Race	− .02	.660
Ethnicity-Hispanic or Latino	.027	.413
Gender	.010	.834
Economic disadvantage	− .012	.741
Location (rural, urban)	.00	.911
Sample composition for studies that report characteristic:^#^		
Race (*n* = 450)		
Asian or Asian American	.00	.898
Black or African American	− .00	.922
Native American or American Indian or Alaska Native	.00	.370
Native Hawaiian or Pacific Islander	.00	.632
White	− .01	.084
Multi-racial/Biracial (must be specified that way)	.00	.803
Not specified	.01	.083
Ethnicity-Hispanic or Latino (n = 375)	.01	.196
Gender-female (*n* = 509)	− .00	.420
Economic disadvantage (n = 168)	.01	.386
Location (n = 426)		
Rural	− .00	.769
Urban	− .00	.455

^*^Logistic regression for binary outcomes; ^#^linear regression for continuous outcomes

Our codes were refined via an iterative process through several pilot tests using five reports not in our sample. Four co-authors (P.B., V.M., A.L., and F.P.) conducted independent reviews and assigned codes. The coding team held five meetings during the pilot testing, one after each report had been reviewed, to discuss the utility of the codes and determine whether adjustments were needed.

### Procedures

To enhance consistency across coders, a glossary was developed and modified following the pilot tests as described above, after which the final set of codes were applied to our sample (see Online Resource Table 3). Studies in our analysis sample were assigned to five coders (which included four of the study’s co-authors; P.B., C.G., A.L., and F.P.). Over 10 separate rounds, 323 (36%) of the 885 programs/studies (including 208 of the 583 U.S. programs/studies, also 36%) were double coded. That is, each report was examined by rotating pairs of individuals on the coding team to ensure consistency and collaboration among all five coders. Coders independently reviewed and coded the reports using an online form. At the end of each round, the resulting pair of codes were compared, and discrepancies were resolved through discussion until reaching consensus among the entire coding team. Interrater reliability was assessed using intraclass correlation coefficients for continuous variables and Cohen’s kappa for dichotomous variables (McHugh, 2012). Online Resource Table 4 reports on the interrater reliability for each measure at the last round of double coding (*n* = 40 programs, 80 ratings). The reliabilities were uniformly acceptable (greater than 0.70) and typically high (over 0.80). Results justified single coding for the remaining 562 programs (64% of the sample) by one member of the team, which met frequently to discuss difficult items - an approach for establishing reliability used in similar studies (e.g., Polo et al., 2019).

### Data Analysis

To answer our primary research questions, we conducted a descriptive analysis on both the main (*n* = 583; i.e., studies conducted within the United States) and full (*n* = 885; i.e., studies conducted within and/or outside of the United States) samples. We answered our secondary research questions using the main analysis sample (*n* = 583) with a series of linear and logistic regressions examining reporting trends over time and chi-square analyses for binary predictors and binary outcomes and linear regression models for binary predictors and continuous outcomes to assess relationships between study quality and (a) culturally tailored programs and (b) reporting.

## Results

Online Resource Table 5 provides study sample characteristics. The studies in the main analysis sample (*n* = 583) included a combination of RCTs (*n* = 258, 44%), cluster RCTs (*n* = 157, 27%), and QEDs (*n* = 168, 29%); most (*n* = 500, 86%) were published in academic journals, commonly targeted elementary, middle, and high school ages, and were delivered in a variety of settings. Over half (*n* = 317, 54%) of the interventions were delivered in schools. Primary outcomes targeted by the interventions included problem behavior (41% of interventions), educational skills and attainment (30%), emotional well-being/mental health (12%), physical health (10%), adult crime (10%), and positive relationships (5%). Sample sizes are presented separately by design in Online Resource Table 6.

Regarding our primary research questions, results of U.S. trials (*n* = 583) showed that 22% of the programs were culturally tailored (i.e., developed to target a specific population; Table 1). Of these, 2% were developed for Black or African American youth, and 4% were developed for Hispanic or Latino youth. Meanwhile, 8% targeted gender (e.g., gender-responsive prevention services for girls with multiple risk factors for juvenile justice system involvement), and 6% targeted youth experiencing economic disadvantage (e.g., individuals within families receiving government assistance). Two percent were developed specifically for urban youth and 1% for rural participants.

Most (but not all) studies reported the racial (77%) and ethnic (64%) characteristics of their samples. Among those that reported race, White (35%) and Black or African American (28%) participants, on average, made up most of the study samples. For approximately one-third of the samples, however, race was conflated with ethnicity or not clearly specified. Hispanic or Latino participants averaged 32% of the samples when ethnicity was reported. It is also worth highlighting that American Indian or Alaskan Native, Asian American, and Native Hawaiian or Pacific Islander populations were largely missing from the samples or combined in a residual “other” category. Gender was frequently reported (87%). The samples were equally divided between the binary categories of male and female; only four studies representing 0.03% of sample participants included another category for non-binary gender identity. Fewer than one-third (29%) of the studies reported participants’ income status, with over half (66%) of the sample participants, on average, considered low income. Of the 73% of studies that described geographic location, 31% included enrollees from rural areas. Patterns detected among the main analysis sample (i.e., studies conducted within the United States) were like those observed in the full sample (i.e., studies conducted within and/or outside of the U.S.; see Online Resource Table 7).

Results of our secondary questions are presented in Table 2 and Online Resource Table 8. Findings from the trend analyses (Table 2) showed no significant increase over time in the reporting of race, ethnicity, gender, income status, or geographic location. One significant downward trend emerged; comparatively, there were fewer programs targeting Black or African American populations in the later years (all 12 studies of programs developed specifically for African American youth were published in the years 2010–2015). When examining the relationship between culturally tailored programs and study quality, results indicated that studies with high internal validity (Steeger et al., 2021; *n* = 65, or 11% of the main analysis sample) were more common for programs that targeted populations from low-income backgrounds and those residing in urban settings, as well as programs with no specific designated target group (Online Resource Table 8). Additionally, high-quality evaluations were more likely to report including populations of low-income. There was no relationship between high-quality evaluations showing beneficial outcomes and programs developed for racial ethnic groups or samples with high proportions of racial ethnic enrollees.

## Discussion

The concept of a syndemic (or multiple pandemics simultaneously) is used to explain the unique, present moment characterized by a mainstream (i.e., White or Northern European descent and US-born) awakening to the nefarious effects of racism (Lee et al., 2022). From this perspective, racial and ethnic disparities in COVID-19 morbidity and mortality rates, coupled with the devastating impacts of environmental injustices and climate change wherein the most severe harms fall disproportionately upon underserved communities, stem from manifestations of systemic racism contributing to social determinants that impact the everyday life experiences of racial ethnic youth and families - or populations presently identified and referred to as racial ethnic minoritized groups in the United States (i.e., individuals who are Asian or Asian American, Black or African American, Native American or American Indian or Alaska Native, Native Hawaiian or Pacific Islander, and Hispanic or Latino). Examples include disinvestment in the community infrastructure in predominantly racial ethnic residential areas, as evident by grocery store and pharmacy closures (Guadamuz et al., 2021; Lee et al., 2020) and unequal access to employment opportunities, affordable and quality healthcare, and safe schools and neighborhoods (EPA, 2021; Lee et al., 2022; Murry et al., 2022). The historic momentum concerning the acknowledgement of these social ills and the need to address racial ethnic inequities served as the impetus for this study. We examined the extent to which intervention programs developed to prevent or reduce internalizing, externalizing or other behavioral problems that hinder a healthy course of youth development and adult maturity have targeted and reported on populations disproportionately impacted by systemic and structural racism, which have been shown to increase risk for both mental and physical health problems. Data from Blueprints for Healthy Youth Development, a registry of preventive behavioral intervention, was used to address questions examining setting and representativeness of racial ethnic populations included in evaluations of these programs.

Results revealed that in a sample of 583 U.S.-based preventive intervention evaluation studies published over an 11-year span (2010–2021), most reported racial (77%) and ethnic (64%) characteristics of their sample, which is considerably higher than other fields. For example, only 43% of U.S. clinical trials of medical treatments reported any race or ethnicity data over the past two decades (Turner et al., 2022). Similarly, Polo et al. (2019) found that over a 36-year period, 43% of RCTs employing psychotherapy and other psychosocial interventions to treat clinical depression reported on participants’ race and ethnicity. Our findings, however, were stable, which contrasts with other studies indicating that the reporting of race and ethnicity has modestly improved over time (Polo et al., 2019; Turner et al., 2022).

An examination of individual-level racial data revealed that, for studies in our sample that reported race, most enrollees were White (35%) followed by Black or African American (28%), and 31% of the enrollees, on average, were collapsed across racial categories as “others” in reference to White versus non-White, or categorized with ethnicity as race, thus ignoring the intersectionality of race and ethnicity. Flanagin et al. (2021) suggest that, while race is a social construct, its social meaning has implications and thus should be reported - though language must be accurate and precise and reflect fairness, equity, and consistency. “Other” as a description for race is uninformative and may be considered derogatory. As such, specific racial and ethnic categories are preferred over collective terms, but if the numbers in some categories are so small, “other” categories should be explicitly defined. Flanagin et al. (2021) also note, “Continual review of the terms and language used in the reporting of race and ethnicity is critically important as societal norms continue to evolve” (p. 621), which could help explain some of our study findings. Indeed, race and ethnicity have salience only to the extent that individuals can self-identify with a particular racial and ethnic identity or that others categorize them with one (Viano & Baker, 2020). For example, Pew Research Center’s 2021 National Survey of Latinos found that respondents may indicate their race as White using the Census Bureau’s method but also indicate their street race as Latino and not White - reflecting the nuances of racial identity, contextual factors, and lived experiences (Pew Research Center, 2021). Racial and ethnic self-identification is also variable, depending on a variety of contextual factors such as macropolitical environment or an individual’s age. As such, both individual identities and racial and ethnic categories are constantly shifting and culture dependent (Liebler et al., 2017; Viano & Baker, 2020). This is reflected in how, from 1790 to 2020, the U.S. Census has used different racial categories every time (Lines et al., 2022).

In addition to race and ethnicity, sociodemographic characteristics such as income and residence should be reported to offer more insight into findings (Flanagin et al., 2021; Gottfredson et al., 2015). We found that most evaluations reported gender within the boundaries of a male–female dichotomy (evenly divided between male and female) and locale (revealing about one-third of studies included participants from rural areas), but less than one-third reported on the representation of participants from low-income backgrounds.

## Implications

Reporting sample characteristics and research contexts is critical to assess heterogeneity (Bryan et al., 2021). A glaring finding of the current study, however, is the omission of consistent and accurate reporting of race and ethnicity in preventive intervention evaluation research, which hinders awareness of the representation of study samples, and if programs are implemented, the potential harms that may occur remain unknown. Moreover, such omission also creates biases in prevention science research evidence (Mohr, 2009). This is a data gap that has also skewed medical evidence (Turner et al., 2022). Further, failing to report race, ethnicity, social demographic characteristic and other relevant contextual descriptors, including whether programs were tested in urban, suburban, and/or rural areas in specific regions of the United States, may conceal disparities, as well as compromise the capacity to determine whether interventions can be recommended to various populations to improve health, development, and overall well-being. This is especially important when information about generalizability does not include specifics regarding how, when, and for whom prevention programs were developed and tested. Addressing these questions should be pursued throughout all stages of design, development, implementation, and evaluation (Murry et al., 2022).

## Recommendations and Future Research

Based on our study findings, we offer several insights for the prevention science field.

### Generalizability of Preventive Intervention Research

Though a clear statement is made about the importance of external validity and analyzing differential effects by subgroups, the evidentiary standards for high quality research endorsed by prevention science focus primarily on internal validity (Gottfredson et al., 2015). We encourage the field to consider adopting external validity standards that provide guidance in evaluating the degree to which preventive intervention research findings are generalizable. In doing so, practices that promote racial and ethnic equity should be integrated as a standard part of conducting high-quality research (Pina et al., 2019; Polo et al., 2019). This could, for example, include a checklist dedicated to racial and ethnic equity that (1) acknowledges the ways in which these guiding principles have been discussed, (2) describes the outcomes of that discussion, and (3) incorporates an internal and external peer procedure to review these concerns. These equity standards could also be used to inform the review process of registries like Blueprints that assess evaluations of interventions according to evidentiary standards historically focused on internal validity (Steeger et al., 2021), thus translating the literature to increase accessibility of evidence-based interventions (Buckley et al., 2020). The large number of registries - up to 24 within the United States and Europe alone (Axford et al., 2022; Burkhardt et al., 2015) - indicates their importance as intermediaries for decision-makers seeking to invest in effective social solutions.

In addition, as more studies of specific preventive interventions are published, of greatest significance are the factors that moderate effectiveness to advance understanding of the reliability of effects across diverse ages, languages, socio-economic backgrounds, and cultures. Research on how to improve youth behavioral outcomes will therefore fall short if it does not explicitly address heterogeneity (Bryan et al., 2021). This means that “what works, under what conditions, and for whom” and “why” must be central questions, and studies need to treat this heterogeneity as a primary rather than secondary concern - an impending focus for some funders (National Academies of Sciences, 2022). In addition, tools such as meta-analysis, which combines independent studies that address a similar question to account for variability in effects and provide reliable estimates of an intervention’s impact (Pigott & Polanin, 2019), or integrative data analysis, which examines multiple raw data sets that have been pooled into one (Kush et al., 2023), can aide in identifying interventions that address the needs of diverse populations. Meanwhile, Evidence and Gap Maps (EGMs), which visually display findings from systematic reviews and impact evaluations in a thematic area, can support evidence-based decision-making by communicating systematic review results in a user-friendly format (Snilstveit et al., 2016). Additional syntheses (like this study) are also needed to identify topics where decision-makers need evidence and where such evidence does not yet exist.

### Cultural Adaptation

We found that 78% of preventive intervention programs in our sample were not developed to target a specific population, indicating they may need to be adapted if/when widely disseminated across diverse settings and populations (Mejia et al., 2017). Any modification to an evidence-based intervention that changes the approach to service delivery, the nature of the therapeutic relationship, or treatment components to accommodate a target population’s cultural beliefs, attitudes, and/or behavior is considered an adaptation (Thier et al., 2020). Thoughtful and deliberate cultural adaptation to improve fit can lead to enhanced engagement, acceptability, and outcomes. However, modifications that remove key elements may be less effective. Using a traffic light as an analogy, Balis and colleagues (2021) assign a color for making changes to an evidence-based intervention: tailoring language or pictures (green/low risk), adding/substituting activities or session sequence (yellow/medium risk), or deleting lessons and decreasing session length (red/high risk). Green light changes improve program fit and could increase cultural appropriateness (e.g., tailoring language and pictures; Balis et al., 2021). For example, Promoting First Relationships® (PFR) is a mental health training program for workers in home-visiting and early care and education settings that has been shown through a well-designed and well-implemented randomized control trial (see Steeger et al., 2021) to promote healthy relationships between caregivers and children from birth to 3 years of age (Oxford et al., 2016). A culturally adapted version was piloted using a randomized control trial (RCT) with American Indian families on a rural reservation (Booth-LaForce et al., 2020). To increase cultural relevance, developers worked with tribal leaders to adopt a relevant name and logo, conducted longer home visits to allow time for conversation, and provided children with a gift at research visits.

As this example illustrates, cultural adaptation should be informed by perspectives of the beneficiaries and responsive to specific cultural contexts, including considerations of language, cultural patterns, and/or values, and can integrate fidelity to components accounting for efficacy while ensuring high cultural relevance (Thier et al., 2020). Since modifications beyond controlled trials are inevitable, practitioners should be guided through training, and supervision to ensure cultural adaptations do not modify core components. Meanwhile, developers and researchers should evaluate which “elements” are “essential” versus modifiable (i.e., those that can be modified without jeopardizing outcomes; Balis et al., 2021). In addition, strategies should be coded to determine which cultural adaptations increase engagement and retention. Meanwhile, mediation and moderation analyses can assess the pathways from cultural adaptation to outcomes as potentially influenced by dimensions of participant responsiveness and whether differences exist across racial and ethnic groups (Barrera et al., 2013, 2017).

### Culturally Tailored Preventive Interventions

Cultural responsiveness is the extent to which a target population’s ethnic and cultural characteristics, experiences, norms, values, and/or behavioral patterns, as well as relevant historical, environmental, and social forces, are incorporated in the design, delivery, and evaluation of targeted health promotion interventions (Jackson, 2009). Top-down adaptations described in the previous section, wherein a practice designed for one group is modified for another group, remain far more common than bottom-up approaches, which account for cultural variation through development within contexts (Hall et al., 2016). Indeed, our results showed a gap in effective culturally responsive preventive interventions developed for racial and ethnic groups. That is, only 22% of preventive interventions evaluated in the United States over the past 11 years targeted specific populations. Of these, 2% were developed for Black or African American youth, and 4% targeted Hispanic or Latino populations. The dearth of culturally tailored programs identified in this review is likely insufficient to mitigate the substantial inequities in schooling, healthcare, substance use treatment, mental health problems, and violence victimization experienced among racial ethnic youth. And compared with cisgender (nontransgender), heterosexual youth, sexual and gender minoritized youth experience even greater inequities, thereby making them a high priority population for culturally tailored programs (Coulter et al., 2019; Mohr, 2009).

There are culturally tailored programs that have met Blueprints’ evidentiary standards assessing the causal validity of RCT and QED studies and dissemination readiness (Buckley et al., 2020; Steeger et al., 2021) that serve as an example for the prevention science field. For example, the *Strong African American Families Program (SAAF)* is listed on the Blueprints registry as showing promising evidence. Adapted from the Blueprints-certified *Strengthening Families Program: For Parents and Youth 10–14* that aims to promote good parenting skills and positive family relationships and reduce aggressive behavior and substance abuse in adolescence (Spoth et al., 2000), SAAF is an interactive program developed to address the unique needs of African American parents and their early adolescent children living in rural communities that has been shown to strengthen family relationships and help teens respond effectively to the risks of substance use, delinquency, and sexual involvement (Brody et al., 2008). Meanwhile, Familias Unidas, which also demonstrates promising evidence following Blueprints’ standards, empowers Hispanic immigrant parents to build a support network and has been shown to help adolescents respond effectively to the risks of substance use and unsafe sexual behavior (Pantin et al., 2009). Future research should study how issues related to discrimination are tackled and how cultural values like familism are leveraged in programs such as SAAF and Familias Unidas to guide the field in developing, testing, replicating, and investing in more culturally tailored programs (Murry et al., 2022) - an effort that is of great importance but beyond the scope of this study.

## Limitations and Conclusions

Our study should be interpreted in the context of some limitations. First, we are the first to review the representation of racial and ethnic groups in behavioral preventive interventions. As such, codes were developed through an iterative process, and missing information and/or unclear descriptions made it difficult to code some studies. Although methods were followed to reach agreement across multiple raters and improve reliability of ratings, human judgment ultimately factored into coding considerations. Second, because not all evaluations reported race and ethnicity, and because we randomly sampled one article from programs with more than one evaluation published after 2010 (which represented 33% of the sample) due to budget and staffing constraints, our findings on sample composition may not generalize to the rest of the Blueprints database. Third, although Blueprints reviews evaluation studies of preventive interventions conducted both nationally and internationally, no robust empirical data exists that specifies in which countries outside of the United States the evaluations were conducted; however, anecdotally, we know that most non-U.S. evaluations were conducted in Europe, Australia, or New Zealand. In addition, other terms for ethnicity may apply within specific nations or ancestry groups outside the U.S. (Flanagin et al., 2021). Therefore, the extent to which our findings represent the world’s prevention intervention trials is unknown. Fourth, we did not code for moderation tests in which studies examined differences in program effects by race, ethnicity, gender, and income. Programs that are not culturally tailored may find that they work better for some groups than others. Such tests are often done on an ad hoc, exploratory basis but may contribute to the understanding of group differences. Given the complexities of analyzing subgroup differences, we did not explore this topic, though it warrants additional study. Fifth, it would have been preferable to include more nuanced categories representing the plethora of terms for describing gender identity and expression. Doing so, however, was not possible since few studies (less than one percent) reported persons of nonbinary gender in their sample - a major limitation. Future research should determine evidence-based strategies for creating inclusive, accurate, and respectful care for all gender identities.

We identified gaps in research on racial and ethnic groups in evaluations of preventive interventions, which calls to action greater awareness of the need to be mindful of potential biases in the prevention science field that are perpetuated when there is an assumption of transferability of programs to populations that have been excluded in the development and testing of the program. Lack of evidence may in fact be an artifact of misalignment because of insufficient information about the study sample and other pertinent contextual factors. We aimed to help build more equal and resilient societies through (1) improved reporting of race, ethnicity, social demographic characteristics, and other relevant contextual descriptors to identify disparities and determine whether preventive interventions can be recommended to various populations; (2) increased investments in the development and testing of culturally responsive preventive interventions for racial and ethnic groups; and (3) guidance in understanding the reliability of effects among different ages, socio-economic backgrounds, and cultures. These efforts will enable effective solutions for improved health and well-being across diverse populations and settings, help reduce disparities, and enhance the credibility and utility of preventive behavioral interventions.

### Supplementary Information

Below is the link to the electronic supplementary material.

Supplementary file1 (DOCX 51 KB)

## Data Availability

Please contact the corresponding author.

## Materials Availability

Materials are included as an online resource.

## Code Availability

Please contact the corresponding author.

## Declarations

### Open Research Statement

This study was not preregistered however a pre-print version is available on Research Square. (10.21203/rs.3.rs-2093401/v1)
