# Supplementary material for: Racial and Ethnic Representation in Preventive Intervention Research: a Methodological Study
Source: Prev Sci. 2023 Jun 29;24(7):1261–74. doi: 10.1007/s11121-023-01564-8 (PMC11161425; doi:10.1007/s11121-023-01564-8)
Supplement: Supplementary file 1 — Supplementary file1 (DOCX 51 KB) [file 11121_2023_1564_MOESM1_ESM.docx]

Racial and Ethnic Representation in Preventive Intervention Research: A Methodological Study

Online Supplement

**Supplemental Panel S1**

***Blueprints inclusion criteria***

The Blueprints database includes evaluations of interventions for youth designed to: (1) prevent or reduce negative behavioral health outcomes (e.g., mental health problems, substance use, delinquency/crime, and other health-related behaviors); or (2) promote positive development (e.g., academic achievement and prosocial behavioral outcomes). The focus on youth limits interventions to those targeting ages 0-24 years, which include post-secondary education and early employment experiences. The one exception comes from interventions designed to reduce recidivism that follow typically young offenders to older ages. Given that the aim of Blueprints is on prevention (including universal, selective, and indicated preventive interventions), the database does not include interventions with a sole focus on evaluating treatment for clinical-level mental health problems, including medical or pharmacological interventions. For each intervention, the database includes experimental evaluations (i.e., RCTs and QEDs but not pre-post designs without a control group) that examine impact (not process evaluations or cost-effectiveness estimates that do not examine condition differences in behavioral outcomes).

***Blueprints Definition of an Intervention and Study***

Blueprints defines a program as an intervention with a pre-specified set of activities, procedures, or treatments that have the goal of improving youth outcomes. Blueprints defines a study as an empirical evaluation of a program using a distinct sample, measures, and methods. Programs in the Blueprints database can have multiple studies, as investigators use different samples and methods to evaluate a program. A study may have one or more reports. Multiple reports within a study examine the same program and use the same sample but cover different outcomes, follow-up periods, and/or mediator or moderator analyses. Blueprints does not examine variations within each report but rather identifies sample characteristics within a study.

**Supplemental Panel S2**

***Search Strategy***

Blueprints uses Boolean operators to create multiple search terms as follows. First, several clauses are used to select journals. Second, search terms are applied to locate outcomes for youth relating to physical and mental health, delinquency, education, prosocial behavior, and problem behavior. Third, these Boolean operators are entered into the Web of Science search engine, which provides subscription-based access to multiple databases with comprehensive citation data for many different disciplines. To locate additional studies, including new evaluations of previously reviewed interventions and new interventions yet to be reviewed, the Blueprints team searches blogs, web pages, other registries, and research organization sites, and accepts nominations from developers and researchers.

**Online Resource Table 1**

***Methods for Search Terms***

| Search term topic | Search Terms/Clauses |
| --- | --- |
| Journals | JOURNAL OF RESEARCH ON EDUCATIONAL EFFECTIVENESS or Adolescence or Aggression and violent behavior or Aggressive behavior or American journal of community psychology or American journal of public health or American journal of sociology or child abuse & neglect or child development or Crime & delinquency or Criminology or Development and psychopathology or Developmental psychology or Deviant behavior or DRUGS-EDUCATION PREVENTION AND POLICY or Evaluation review or Future of children or Journal of adolescence or Journal of adolescent health or Journal of adolescent research or Journal of applied developmental psychology or Journal of child & adolescent substance abuse or Journal of child psychology and psychiatry and allied disciplines Educational Evaluation and Policy Analysis or American Educational Research Journal or Review of Educational Research or Journal of community psychology or Journal of consulting and clinical psychology or Journal of criminal justice or JOURNAL OF EDUCATIONAL AND PSYCHOLOGICAL CONSULTATION or Journal of family therapy or Journal of family violence or Journal of interpersonal violence or Journal of marriage and the family or JOURNAL OF RESEARCH IN CRIME AND DELINQUENCY or journal of research on adolescence or journal of school psychology or journal of studies on alcohol or JOURNAL OF THE AMERICAN MEDICAL ASSOCIATION or MERRILL-PALMER QUARTERLY-JOURNAL OF DEVELOPMENTAL PSYCHOLOGY or Preventive medicine or Psychological bulletin or School psychology review or Youth & society |
| Mental health | *(program or evaluation or prevention or intervention) and (depression or anxiety or suicide or PTSD or POSTTRAUMATIC-STRESS-DISORDER or obesity or “physical health”) not adult* |
| Delinquency | *(program or evaluation or prevention or intervention) and (violence or aggression or “conduct problem*” or “substance use” or delinquency or “teen* pregnancy” or sex* or bullying or “child maltreatment” or gang or truancy or victimization or school) not adult* |
| Education | *(program or evaluation or prevention or intervention) and (academic or employment or cognitive develop* or dropout or post-secondary or vocational) and (school or community or criminal justice)* |

**Online Resource Table 2**

Codebook

| Field | Code |
| --- | --- |
| Group(s) targeted by the program (select all that apply)^a^ | Asian or Asian American  Black or African American  Native American or American Indian or Alaska Native  Native Hawaiian or Pacific Islander  White  Hispanic or Latino  Gender  Youth in rural communities  Youth in urban communities  Youth/families that are low-income  Individuals in foster care or child welfare system  No group explicitly targeted |
| Age (select all that apply)^a^ | Infant (0-2)  Early childhood (3-4) – preschool  Late childhood (5-11) – kindergarten/elementary  Early adolescence (12-14) – middle school  Late adolescence (15-18) – high school  Early adulthood (19-24)  Adult |
| Primary place of the intervention^a^ | Community  Correctional facility/community corrections/court  Home  Online  Mental health/treatment center/hospital/medical center  School  Social services |
| Targeted primary outcome(s) (select all that apply, but try to identify one)^a^ | Adult crime  Educational skills and attainment  Emotional well-being/mental health  Physical health  Positive relationships  Problem behavior |
| Blueprints-certified^[[1]](#footnote-1)^ | Yes/No |

| Field, continued | Code, continued |
| --- | --- |
| Study design | Cluster Randomized Control Trial (c-RCT)  Quasi-Experimental Design (QED)  Randomized Control Trial (RCT) |
| Published in a refereed journal | Yes/No |
| Country where the study was conducted (select all that apply) | United States  Outside of the United States |
| Locale where the study was conducted (select all that apply) | Rural  Suburban  Urban  N/A (conducted solely outside the USA or conducted within the U.S. and locale not reported or not clear) |
| Region where the study was conducted (select all that apply) | Northeast  Midwest  South  West  United States Territories  N/A (conducted solely outside the US or not reported) |
| Overall study sample size | Individual N, Cluster N (for c-RCT only) |
| Reported racial data | Yes/No |
| Racial composition* | % Asian or Asian American (0-100)  % Black or African American (0-100)  % Native American or American Indian or Alaska Native (0-100)  % Native Hawaiian or Pacific Islander (0-100)  % White (0-100)  % Biracial or multi-racial (0-100)  % Not specified (0-100) |
| Reported ethnicity data | Yes/No |
| Ethnic composition* | % Hispanic or Latino (0-100)  % Not Hispanic or Latino (0-100) |
| Reported gender data | Yes/No |
| Gender composition* | % Male (0-100)  % Female (0-100)  % Other (0-100) |
| Reported economic disadvantage data | Yes/No |
| Income composition* | % Economically disadvantaged (0-100) |

Note: *Branching logic was used to skip this question if a coder responded "no" to the previous question. ^a^Program-level codes (all other fields were coded at the study level).

**Supplemental Panel S3**

***Intervention Characteristics: Specific Population(s) for which the Program Was Developed***

In terms of intervention characteristics, we sought to determine if the programs in our sample were specifically developed for certain populations based on race, ethnicity, gender, economic disadvantage, and/or geographic location (i.e., urban vs. rural). A group was considered “explicitly targeted” only if the group was part of the program name and/or if the program description clearly specified that the intervention was developed for that group. If applicable, coders could select multiple targeted groups.

***Sample Characteristics: Race, Ethnicity, Gender, and Economic Disadvantage***

For the sample characteristics, we drafted two sets of codes for each variable. The first set were dichotomous (yes/no) codes used to indicate whether a given study reported individual-level racial, ethnicity, gender, and income data for the sample. The second set of codes, described in more detail below, were used to assess sample composition.

For the initial racial and ethnic composition codes, we followed U.S. Census Bureau coding operations, which treat race and ethnicity as separate identities. Concerning the reporting of race, Census guidelines require the following five categories at minimum: (1) Asian or Asian American; (2) Black or African American; (3) Native American or American Indian or Alaska Native; (4) Native Hawaiian or Pacific Islander; and (5) White (Humes, Jones, & Ramirez, 2011). We added “percent not specified” to our list of racial codes to account for studies that combined two or more racial categories (e.g., Asian and Pacific Islander, a residual other category) or used more detailed classifications not captured by the five Census codes. It should also be noted that the proportion of the sample identifying as Hispanic or Latino was coded as “not specified” for race, as that value was entered into the subsequent ethnicity field. “Percent biracial/multi-racial” was added after our pilot test to account for individuals identifying with multiple races (but not ethnicities).

For ethnicity, two categories (i.e., “Hispanic or Latino” or “not Hispanic or Latino”) was the minimum requirement according to current Census policies (Humes et al., 2011). The codes for gender were: (1) male; (2) female; and (3) persons of nonbinary gender. When coding for race, ethnicity, and gender composition, we entered the proportion of the sample identifying with each of the possible categories.

Finally, to code for economic disadvantage, we opted against relying on income means, medians, or distributions. Instead, to ease interpretation, we considered four proxies: (1) qualifies for the free/reduced-price lunch program; (2) receives Medicaid; (3) Pell-eligible; and (4) qualifies for the Children’s Health Insurance Program. We also considered the proportion of the sample that fell within the federal government’s poverty level, and the lowest income category of an income distribution. We then coded the proportion of the sample fitting with any of these proxies used to indicate economic disadvantage. However, the sample proportions classified as low socioeconomic status (SES) were coded as “not reported” for economic disadvantage, since SES is typically measured as a combination of education, occupation, and income^[[2]](#footnote-2)^.

***Setting Characteristics: Locale***

For the setting characteristics, we were especially interested in the locale where the study was conducted. We focused on urbanicity with rural, suburban, and urban as initial options. Coders were instructed to choose a setting based on the author’s description, and coders could choose more than one type of locale if the study was conducted in multiple settings.

**Online Resource Table 3**

Glossary

| Code | Notes |
| --- | --- |
| Age | - For programs aimed at reducing recidivism among adult offenders, mark “adult.” - For all other age groups, ages are approximations of school level. - If a program targets a specific school level, use the school categories from the program description even if sample ages fall into the adjacent categories. For example, code only Early Adolescence (12-14) – Middle School for middle school programs for which the sample might include adolescents ages 11 or 15. - If the program does not target a specific school level, be inclusive across all categories, i.e., if a program targets 5- to 12-year-olds, check Late Childhood (5-11) – K/Elementary and Early Adolescence (12-14) – Middle School. |
| Primary Place of Intervention | - If the intervention is evenly implemented in more than one place, go to more personal first (e.g., home first, school second, community third). |
| Study-level codes | - Only code one report per study; if multiple reports within one study/sample exist (e.g., posttest, moderation/mediation, long-term follow-up, etc.), code the initial RCT/QED (i.e., reports the posttest). - If more than one sample is discussed within a report, and the same design is used, average the demographics across samples. - If a report includes two samples and one sample was evaluated with a RCT and the other with a QED, code the demographics from the RCT (i.e., the stronger study). - If the report *only* provides demographic information for the family or parent and not specifically for the child, record “no” for whether racial, economic, gender and economic information is reported. However, if the report is not clear whether data represent the family *or* explicitly the child only, record the demographic percentages. - Rely only on the article itself (or online supplementary materials referenced in the main article) without consulting other references. |
| Study design | - An RCT if there is one child analyzed per family (i.e., family *n* = child *n*) - A CRT if there is more than one child analyzed in any of the families (i.e., family *n* < child *n*); the number of families equals the cluster *n* and the number of children equals the individual *n*. - Quasi-experimental studies include interrupted time series, regression discontinuity, propensity score matching, limited or no matching. |
| Locale where the study was conducted | - Choose multiple types of locales if the study was conducted in multiple settings. - If the study was conducted statewide or nationwide, assume it was tested in rural, suburban, and urban areas. - If the study was conducted at the county level and the locale is not clear, check N/A. - If only cities are named, check Urban. If “city” is the only description, check Urban. - If mid-sized or large town is the description, mark as Urban. If only description is small town, mark as rural. - If the only description is that the study was conducted in a “metropolitan area,” code Suburban, Urban. Or, if the only description includes a city name (or names) and the word “area,” code Suburban, Urban. - If the only term used is “semi-urban,” code as Urban. |
| Sample size | - Only add cluster sample size if this is a cluster RCT. Ignore cluster size for QEDs. - If both the randomized and analysis sample sizes are reported, code the sample size for which the demographic figures are reported. For example, use the sample size reported in the same table that reports the demographic information you are using to code race, ethnicity, gender, and/or economic disadvantage data. - If a table with sample descriptives and sample size is not provided, look for a CONSORT diagram. If neither are available, read the text. - If you do not know from tables or the text which sample (randomized or analysis) is reported and you read two different sample sizes in the text, pick the analysis sample (i.e., the smaller number). However, if different sample sizes are presented for different outcomes, take the average of the different figures. |
| Reported racial data | - If only the school, district, or city racial data from which the sample was obtained is reported (and not the individual-level sample racial data) code “No.” - If the intervention targets a specific racial group (e.g., Black children), but the individual-level racial data for the sample is unclear (i.e., the report does not explicitly state that the sample selected only Black children or that all sample participants were black), code “No.” - If 100% end up “Not Specified” (e.g., 100% of the sample is Latino/Hispanic), then code “No.” - Multiple races/ethnicities (meaning race and ethnicity is combined into one category) is coded as a “yes” for “reported race” and “not specified” for “racial composition” unless there is no other racial information, in which case code as “No.” Make sure any “multiple” or combination does not include “ethnicity” so that “multi-racial” is limited to races (to be coded as “multi-racial”). - If one of the race categories is listed as non-Hispanic (e.g., “non-Hispanic White”), because this is vague and race is combined with ethnicity, code “No.” - If the study only reports something related to “% Minority” and gives a percentage, code that percentage as “Not Specified” under “race” for racial composition and the residual as “White” (also for racial composition). |
| Racial composition | - If a study combines racial categories (e.g., percentage of Asian and Pacific Islander students) for reporting, then record those percentages as “Not Specified.” However, if Asian is stated alone, assume Pacific Islanders are not included and code in the %Asian or Asian American category. - Latino/Hispanic coded as “Not Specified.” However, if counting “Hispanic” as “Not Specified” creates a summed total greater than 100%, calculate the residual for race without including Hispanics. - “Multi-ethnic” or “multiple race/ethnicity” coded as “Not Specified.” - If the study is conducted outside of the U.S., code racial composition using U.S. Census codes. No adaptations can be made for other countries. For example, if the study was conducted in Australia with “native aboriginals,” code as “Not Specified” for race. Or, if the study states the percentage of German and non-German, code as “Not Specified.” |
| Reported ethnicity data | - The study must explicitly report “Hispanic” or “Latino” to get a “Yes.” - List of samples from Hispanic or Latino countries of Central and South America counts as Hispanic. - If only the school, district, or city ethnic data from which the sample was obtained is reported (and not the individual-level sample ethnic data), code “No.” - If the intervention targets a specific ethnic group (e.g., Spanish-speaking children in a US public school), but individual-level ethnic data for the sample is unclear (i.e., the report does not explicitly state that the sample selected only Hispanic children or that all sample participants were Hispanic), code “No.” |
| Ethnic composition | - The US Census defines "Hispanic or Latino" as a person of Cuban, Mexican, Puerto Rican, South or Central American, or other Spanish culture or origin regardless of race. “Not Hispanic or Latino” is the residual. |
| Reported gender data | - If only the school, district, or city gender data from which the sample was obtained is reported (and not the individual-level sample gender data), code “No.” - If the intervention targets a specific gender (e.g., Males), but individual-level gender data for the sample is unclear (i.e., the report does not explicitly state that the sample selected only males or all sample participants were male), code “No.” |
| Reported economic disadvantage data | - Proxies for low-income status and/or families living in poverty (i.e., economic disadvantage) include: Qualifies for the free/reduced lunch (FRL) program; Receives Medicaid; Pell-Eligible; Qualifies for the Children's Health Insurance Program (CHIP). - If only the school, district, or city data from which the sample was obtained is reported (and not the individual-level sample data), code “No.” - If the intervention targets a specific economic group (e.g., Head Start children), but individual-level poverty data is not reported (i.e., did not specify the sample included only low-income participants), code “No.” - Unless otherwise specified, SES is an incomplete measure of income status and should be coded as “No.” |

**Online Resource Table 4**

Inter-rater reliabilities: Last Round of Double-Rated Programs (*n* = 40 programs, 80 ratings)

| Categorical Variables | Kappa |
| --- | --- |
| Group(s) targeted by program  Age  Primary place of the intervention  Targeted primary outcome  Study design  Country where study was conducted  Locale where study was conducted  Region where study was conducted  Reported race data  Reported ethnicity data  Reported gender data  Reported economic disadvantaged data  Published in refereed journal | 1.0  .86  .90  .93  .96  1.0  .87  .91  .95  1.0  .89  1.0  1.0 |

| Continuous Variables | ICC |
| --- | --- |
| Individual sample size  Cluster sample size  % Asian or Asian American  % Black or African American  % Native American or American Indian  % Native Hawaiian or Pacific Islander  % White  % Multi-Racial or Biracial  % Race not specified  % Hispanic or Latino  % Male  % Economically Disadvantaged | .98  .74  1.0  .99  1.0  ---  .83  1.0  .70  .99  .99  .99 |

**Online Resource Table 5**

Descriptive Statistics for Preventive Intervention and Evaluation Study Characteristics

|  | Sample | |
| --- | --- | --- |
|  | Full (n=885)  n (proportion) | U.S. (n=583)  n (proportion) |
| Design  Cluster randomized control trial (c-RCT)  Quasi-experimental design (QED)  Randomized control trial (RCT)  Article published in an academic journal  Blueprints-certified study (well-designed, well-implemented; high internal validity; Steeger et al., 2021)  Primary age group targeted by the preventive intervention*  Infant (ages 0-2 years)  Preschool (ages 3-4 years)  Elementary school (ages 5-11 years)  Middle school (ages 12-14 years)  High school (ages 15-18 years)  Young adult (ages 19-24 years)  Adult  Setting  Community  Correctional facility  Home  Hospital, medical center  Online  School  Social services  Primary outcome targeted by the preventive intervention*  Adult crime (recidivism)  Educational skills and attainment  Emotional well-being, mental health  Physical health  Positive relationships  Problem behavior | 274 (.31)  251 (.28)  360 (.41)  785 (.89)  80 (.09)  57 (.06)  139 (.16)  353 (.40)  338 (.38)  276 (.31)  88 (.10)  72 (.08)  107 (.12)  87 (.10)  79 (.09)  58 (.07)  30 (.03)  511 (.58)  13 (.02)  72 (.08)  227 (.26)  140 (.16)  98 (.11)  50 (.06)  363 (.41) | 157 (.27)  168 (.29)  258 (.44)  500 (.86)  65 (.11)  37 (.06)  87 (.15)  212 (.36)  220 (.38)  189 (.32)  70 (.12)  57 (.10)  71 (.12)  69 (.12)  55 (.09)  42 (.07)  20 (.03)  317 (.54)  9 (.02)  58 (.10)  176 (.30)  69 (.12)  59 (.10)  27 (.05)  237 (.41) |

Note: *Percentages add to more than 100, as programs may target multiple groups and multiple outcomes.

**Online Resource Table 6**

Average Sample Sizes for Evaluations of Preventive Interventions: By Sample and Experimental Design

|  | Individuals: All Designs | | Clusters: c-RCTs | | Individuals: c-RCTs | | Individuals: QEDs | | Individuals: RCTs | |
| --- | --- | --- | --- | --- | --- | --- | --- | --- | --- | --- |
|  | Full | US | Full | US | Full | US | Full | US | Full | US |
| N  Mean  Median  Std. Dev.  Minimum  Maximum | 885  2,407  409  13,501  12  326,987 | 583  2,975  440  16,400  12  326,987 | 268  42  25  48  2  418 | 153  44  27  45  2  250 | 274  2,356  940  6,194  48  89,707 | 157  2,847  942  7,881  51  81,707 | 251  4,968  632  24,217  12  326,987 | 168  6,431  782  29,199  12  326,987 | 360  661  200  1,896  16  23,133 | 258  803  236  2,206  18  23,133 |

Note: c-RCT – Cluster Randomized Control Trial; RCT – Randomized Control Trial; QED – Quasi-Experimental Design.

**Online Resource Table 7**

Descriptive Results (Full Sample of Studies conducted within and outside the United States)

| Interventions developed for a specific population (total n = 885):*  Race  Asian or Asian American  Black or African American  Native American or American Indian or Alaska Native  Native Hawaiian or Pacific Islander  White  Ethnicity  Hispanic or Latino  Gender  Economic disadvantage  Location  Rural  Urban  No specific group  Studies that reported sample distribution of (total n = 885):  Race  Ethnicity – Hispanic or Latino  Gender  Economic disadvantage  Location (rural, urban)  Sample statistics for studies that report characteristic:  Race (n = 504)  Asian or Asian American  Black or African American  Native American or American Indian or Alaska Native  Native Hawaiian or Pacific Islander  White  Multi-racial/Biracial (must be specified that way)  Not specified  Ethnicity – Hispanic or Latino (n = 380)  Gender – Female (n = 764)^a^  Economic disadvantage (n = 198)  Location (n = 428)  Rural  Urban | **N (Proportion)**  6 (.01)  12 (.01)  5 (.01)  1 (.00)  0 (.00)  26 (.03)  58 (.07)  39 (.04)  3 (.00)  10 (.01)  728 (.82)  504 (.57)  380 (.43)  764 (.86)  198 (.22)  428 (.48)  **Mean (SD)**  .04 (.11)  .26 (.28)  .01 (.09)  .00 (.01)  .38 (.30)  .01 (.04)  .30 (.26)  .32 (.27)  .48 (.21)  .61 (.27)  .31 (.46)  .89 (.31) |
| --- | --- |

Notes: *Percentages add to more than 100, as interventions may target multiple groups. ^a^Four studies reported another category for persons of nonbinary gender, which averages .03% of the sample participants.

**Online Resource Table 8**

Results of “Certified” High Internal Validity Studies (Steeger et al., 2021) (U.S. studies)

| Outcomes | Coefficient for  Certification Measure | *p* |
| --- | --- | --- |
| Developed for a specific population (n = 583):^a^  Race  Asian or Asian American  Black or African American  Native American or American Indian or Alaska Native  Native Hawaiian or Pacific Islander  White  Ethnicity  Hispanic or Latino  Gender  Economic disadvantage  Location  Rural  Urban  No specific group  Studies that reported sample distribution of (n = 583):^a^  Race  Ethnicity – Hispanic or Latino  Gender  Economic disadvantage  Location (rural, urban)  Sample composition for studies that report characteristic:^b^  Race (n = 450)  Asian or Asian American  Black or African American  Native American or American Indian or Alaska Native  Native Hawaiian or Pacific Islander  White  Multi-racial/Biracial (must be specified that way)  Not specified  Ethnicity – Hispanic or Latino (n = 375)  Gender – Female (n = 509)  Economic disadvantage (n = 168)  Location (n = 426)  Rural  Urban | 0.25  2.37  0.63  0.13  ---  0.05  0.30  9.85*  0.80  9.38^*^  9.57^*^  0.07  2.03  0.79  8.90^*^  0.20  -.01  .08  -.01  .00  -.03  -.00  -.03  -.05  .04  .06  .04  .05 | .616  .123  .426  .723  ---  .830  .586  .002  .370  .002  .002  .795  .154  .374  .003  .655  .699  .059  .377  .982  .483  .876  .426  .221  .214  .232  .605  .305 |

Note: ^a^Chi-square value for binary predictor and binary outcomes; ^b^Linear regression models for binary predictor and continuous outcomes. *Statistically significant (*p* < .05).

1. High internal validity (for a definition, see: Steeger, C. M., Buckley, P. R., Pampel, F. C., Gust, C., & Hill, K. G. Common methodological problems in randomized controlled trials of preventive interventions. *Prevention Science, 22*(8), 1159-1172. <https://doi.org/10.1007/s11121-021-01263-2>). [↑](#footnote-ref-1)
2. Mueller, C. W., & Parcel, T. L. (1981). Measures of socioeconomic status: Alternatives and recommendations. *Child development*, 13-30. https://doi.org/10.2307/1129211 [↑](#footnote-ref-2)
